# Supplementary material for: A New Computer Model for Evaluating the Selective Binding Affinity of Phenylalkylamines to T-Type Ca2+ Channels
Source: Pharmaceuticals (Basel). 2021 Feb 10;14(2):141. doi: 10.3390/ph14020141 (PMC7916697; doi:10.3390/ph14020141)
Supplement: Supplementary file 1 [file pharmaceuticals-14-00141-s001.pdf]

Supplementary Material

Supplementary Tables and Figures

1.1 Supplementary Tables

Supplementary Table S1. Numerical parameter settings for running PyGBe.

Parameters	Value	Notes
K	1	Gauss points per elements
NK	5	Gauss points per triangle edge
K_{fine}	19	Gauss points per element for near singular integrals
Threshold	0.8	the threshold to stop integration while near singularity
BSZ	200	CUDA block size
restart	100	number of iterations for GMERS to do the restart
tolerance	1e-6	GMER convergence tolerance
max_{iter}	5000	Max. number of GMERS iterations run
P	2	order of expansion in tree code
eps	1e-12	machine error
NCRIT	500	max number of boundary elements per twig box
θ	0.66	Multipole acceptance criterion of treecode
GPU	1	0: not use GPU, 1: use GPU

Supplementary Table S2. Normality test for P-loop remodeling data (α_1G) from two groups with different sampling sizes.

Domain ID	Anderson-Darling Normality Test Critical Value	Significance Level	Statistic Results (500)	Statistic Results (20,000)	Kruskal-Wallis One-way ANOVA 500 vs 20,000
1	0.781	5	5.712	225.364	0.987
2	0.781	5	22.217	1090.299	0.868
3	0.781	5	11.096	335.999	0.497
4	0.781	5	12.014	506.270	0.984

Supplementary Table S3. The structures and properties of computer-designed compounds using Deep-Learning (D: distance; C: count; F: frequency).

	SMILE	D	C	F	logP	QED	SAS
0	COc1ccc(CCCOC(Oc2ccno2)c2ccc(OC)cc2)cc1	4.05	23	0.52	3.27	0.51	3.16
1	COc1ccc(CCCOC(Oc2ccoc2)c2ccc(OC)cc2)cc1	4.38	4	0.09	0.96	0.61	2.90
2	COc1ccc(CCC(CCOc2ccno2)c2ccc(OC)cc2)cc1	4.42	2	0.04	1.04	0.60	2.37
3	COc1ccc(CC(COCOc2ccno2)c2ccc(OC)cc2)cc1	4.44	1	0.02	2.28	0.91	3.45
4	COc1ccc(CCOC(COc2ccno2)c2ccc(OC)cc2)cc1	4.52	2	0.04	2.45	0.84	2.67

Supplementary Table S4. Predicted binding affinity (K_d) by Vina versus experimental measurement of IC_{50} of given TCC blockers (unit: micromolar).

	Mibefradil (Pred./Exp.)	NNC 55-0395 (Pred./Exp.)	NNC 55-0396 (Pred./Exp.)	NNC 55-0397 (Pred./Exp.)	RO 40-5966 (Pred./Exp.)	SKF-96365 (Pred./Exp.)
α_1C	30.57/21.0[1]	58.54/100[2]	0/N.A.	30.57/100[2]	95.31/0.865[3]	0/N.A.
α_1G	4.35/1.2[4]	25.99/64[5]	1.93/6.8[5]	6.02/8[5]	7.08/10.08[5]	112.12/0.56[6]
α_1H	6.02/1.2[4]	22.09/N.A.	3.7/10[7]	11.53/N.A.	6.02/N.A.	155.15/0.56[6]
α_1I	25.99/2.3[4]	22.09/N.A.	3.7/N.A.	5.12/N.A.	13.57/N.A.	0/0.56[6]

1.2 Supplementary Figures

Supplementary Figure S1. The negatively charged lysine affects the electric potential distribution of aspartic acid in the x-y plane. (A) The Dist. of E_D (0,0) without K; (B) The Dist. of E_D (0,0) after adding the lysine E_K (3.8, 0).

Supplementary Figure S2. The predicted versus theoretical RMSD plots for group sampling size 500 and 20,000. (A,B,C,D) Domains I to IV for 500 sampling size group; (E,F,G,H) Domains I to IV for 20,000 sampling size group.

Supplementary Figure S3. The predicted 3D binding plots between TC 7 and α_1G. An alkyl bond (4.31 angstroms) has been formed between TC 7 (green) and the sidechain of V^{1i24} (red) at domain I. The sidechain of E^{1p50} is colored as blue and one Ca^{2+} is colored as yellow.

Supplementary Figure S4. Based on the 2D structure of SKF-96365, 10 structures have been found using the Deep-Learning based de novo drug design approach.

Supplementary Figure S5. 2D structure and chemical properties of the redesigned phenylalkylamine analog, TC 1.

Supplementary Figure S6. 2D structure and chemical properties of the redesigned phenylalkylamine analog, TC 2.

Supplementary Figure S7. 2D structure and chemical properties of the redesigned phenylalkylamine analog, TC 3.

Supplementary Figure S8. 2D structure and chemical properties of the redesigned phenylalkylamine analog, TC 4.

Supplementary Figure S9. 2D structure and chemical properties of the redesigned phenylalkylamine analog, TC 5.

Supplementary Figure S10. 2D structure and chemical properties of the redesigned phenylalkylamine analog, TC 6.

Supplementary Figure S11. 2D structure and chemical properties of the redesigned phenylalkylamine analog, TC 7.

Supplementary Figure S12. 2D structure and chemical properties of the redesigned phenylalkylamine analog, TC 8.

Supplementary Figure S13. 2D structure and chemical properties of the redesigned phenylalkylamine analog, TC 10.

Supplementary Figure S14. 2D structure and chemical properties of the redesigned phenylalkylamine analog, TC 11.

Supplementary Figure S15. 2D structure and chemical properties of the redesigned phenylalkylamine analog, TC 12.

Supplementary Figure S16. 2D structure and chemical properties of the redesigned phenylalkylamine analog, TC 13.

Supplementary Figure S17. 2D structure and chemical properties of the redesigned phenylalkylamine analog, TC 15.

Supplementary Figure S2

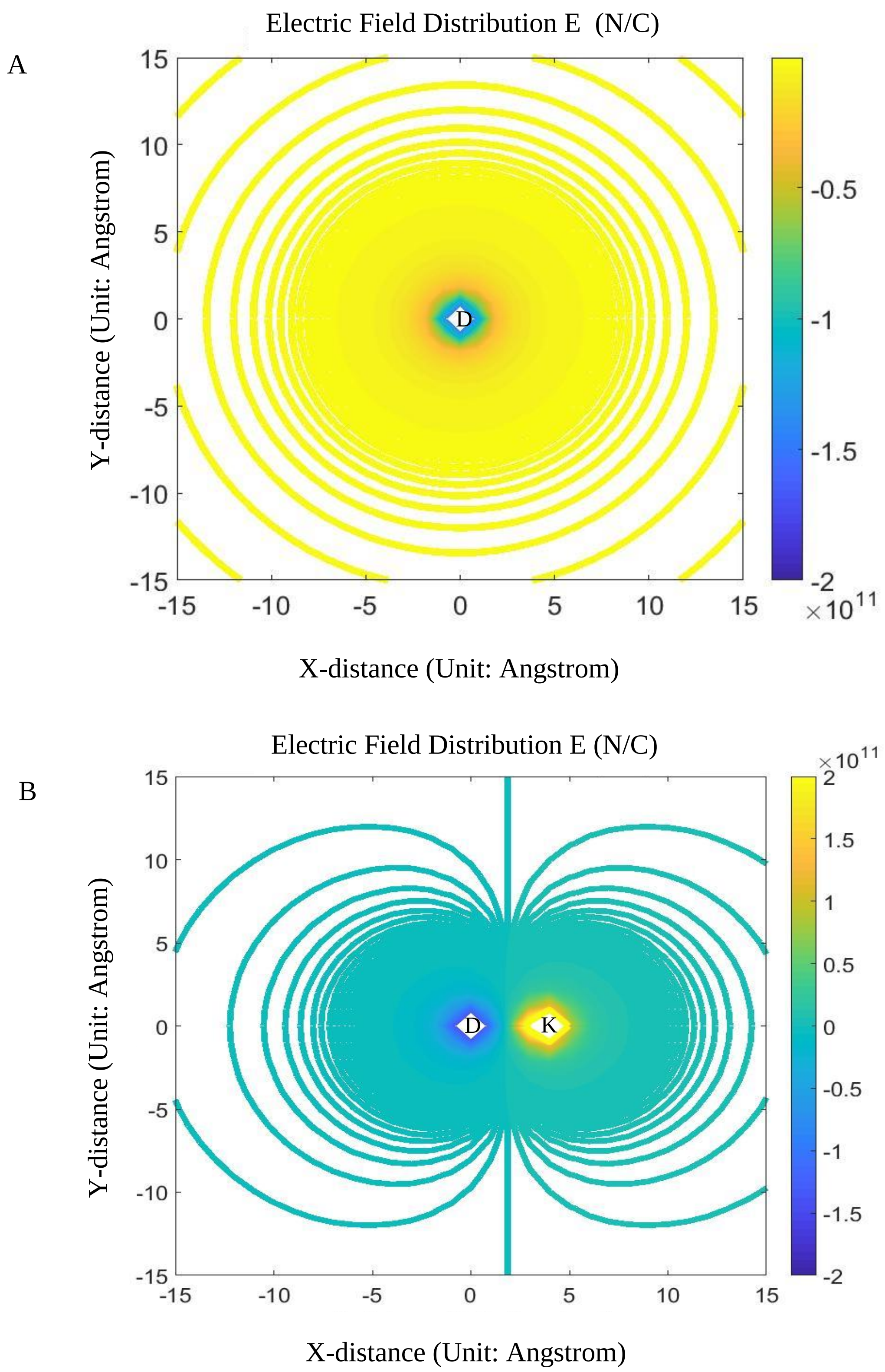

Supplementary Figure S2

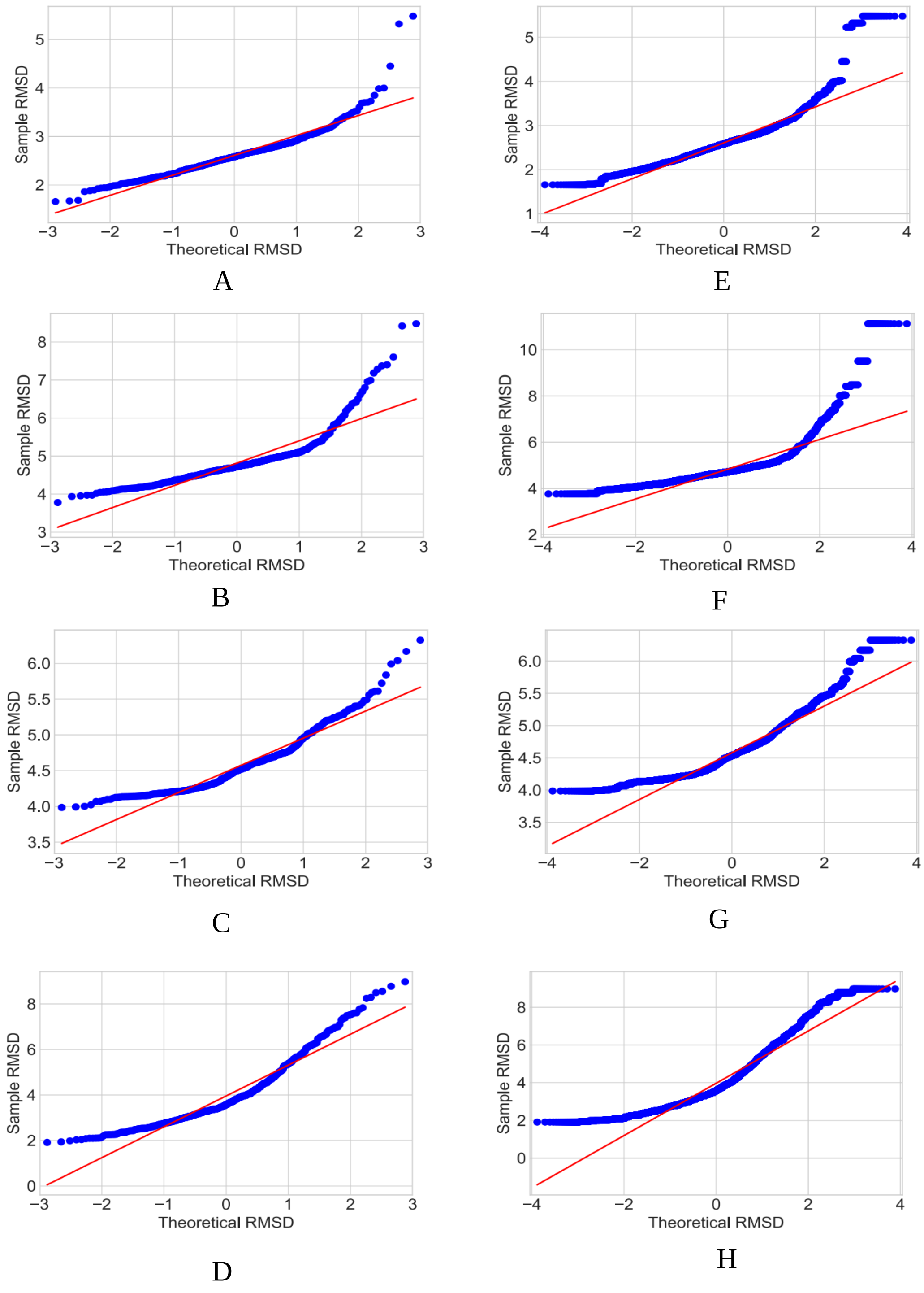

Supplementary Figure S3

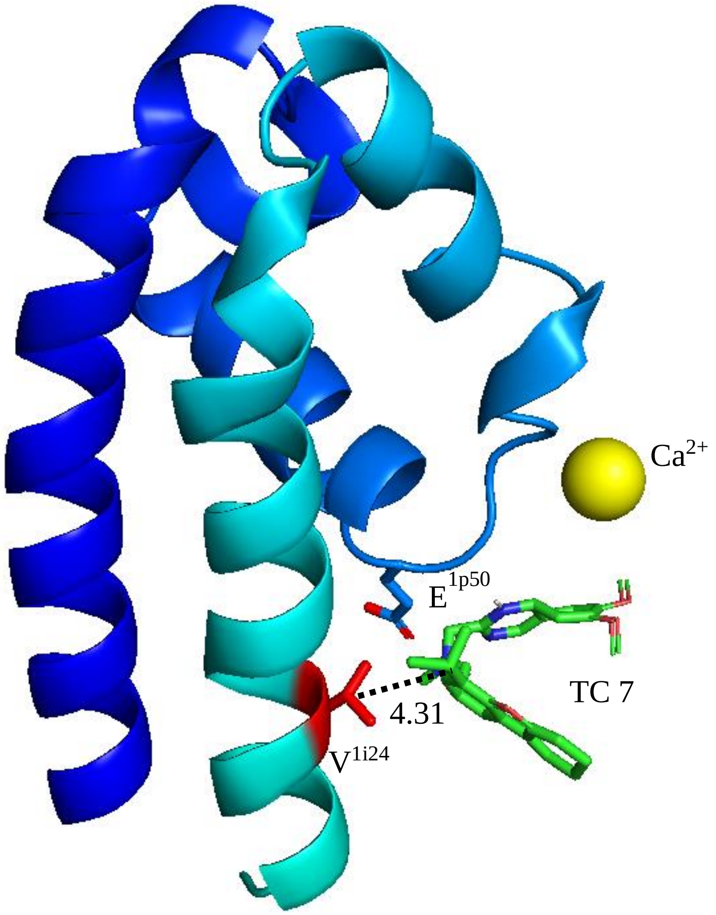

Supplementary Figure S4

SKF-96365

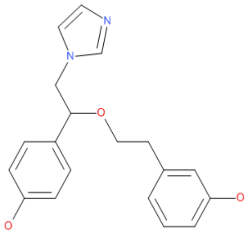

Analog 1

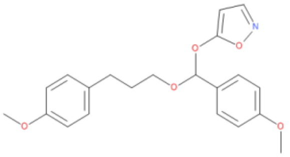

Analog 2

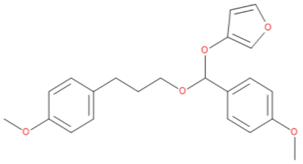

Analog 3

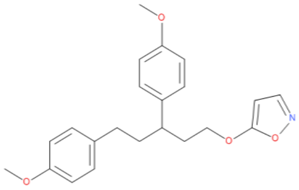

Analog 4

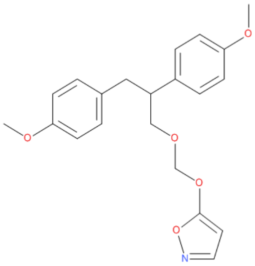

Analog 5

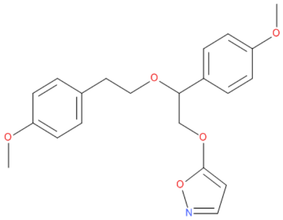

Analog 6

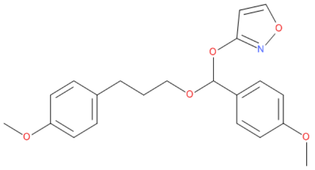

Analog 7

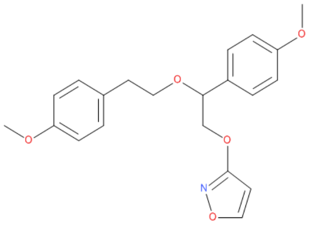

Analog 8

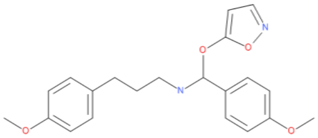

Analog 9

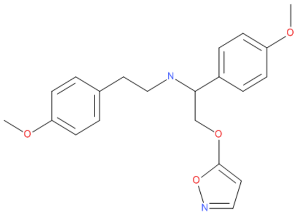

Analog 10

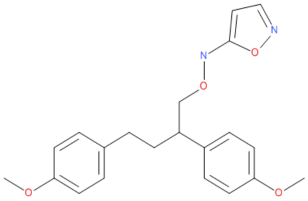

Analog 11

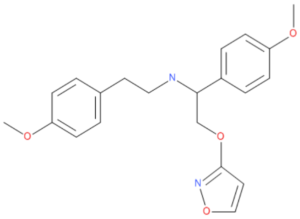

Supplementary Figure S5

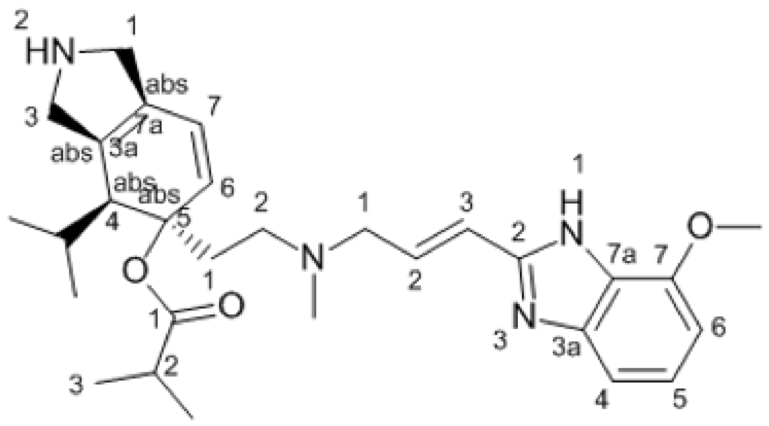

(4S,5R)-4-isopropyl-5-2-(((E-3-(8-methoxy-1H-benzo[d]imidazole-2-yl)allyl)(methyl)amino)ethyl)-2,3,4,5-tetrahydro-1H-isoindol-5-yl isobutyrate

PSA: 79.47

ALogP: 4.5079

Stereo Center Count: 2

Hydrogen Acceptor Count: 6

Hydrogen Donor Count: 2

Composition: C: 70.7% H: 8.2% N: 11.4% O: 9.7%

Formula Weight: 492.6529

Exact Mass: 492.31004118

Molecular Formula: $C_{29}H_{40}N_4O_3$

Supplementary Figure S6

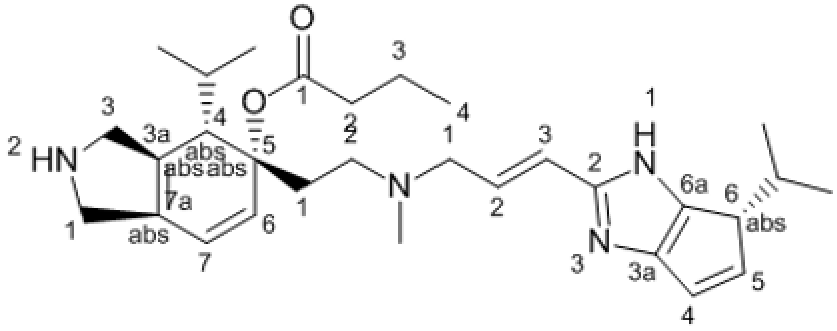

(4S,5R)-4-isopropyl-5-2-(((E-3-(R-6-isopropyl-1,6-dihydrocyclopenta[d]imidazole-2-yl)allyl)(methyl)amino)ethyl)-2,3,4,5-tetrahydro-1H-isoindol-5-yl butyrate

PSA: 70.25

ALogP: 4.9711

Stereo Center Count: 3

Hydrogen Acceptor Count: 5

Hydrogen Donor Count: 2

Composition: C: 73.1% H: 9% N: 11.4% O: 6.5%

Formula Weight: 492.69596

Exact Mass: 492.346426688

Molecular Formula: $C_{30}H_{44}N_4O_2$

Supplementary Figure S7

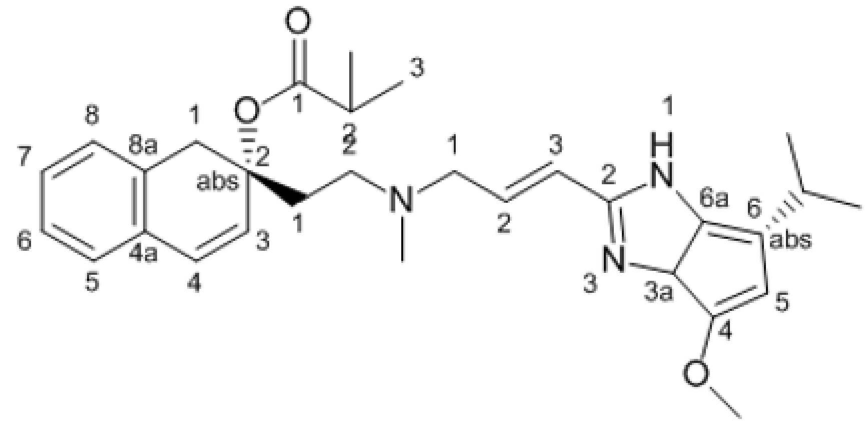

(2R)-2-(2((E-3-(6-isopropyl-4-methoxy-1,3a-dihydrocyclopenta[d]imidazole-2-yl)allyl)(methyl)amino)ethyl)-1,2-dihydronaphthalen-2-yl isobutyrate

PSA: 63.16

ALogP: 4.4401

Stereo Center Count: 2

Hydrogen Acceptor Count: 6

Hydrogen Donor Count: 1

Composition: C: 73.6% H: 8% N: 8.6% O: 9.8%

Formula Weight: 489.64896

Exact Mass: 489.299142138

Molecular Formula: $C_{30}H_{39}N_3O_3$

Supplementary Figure S8

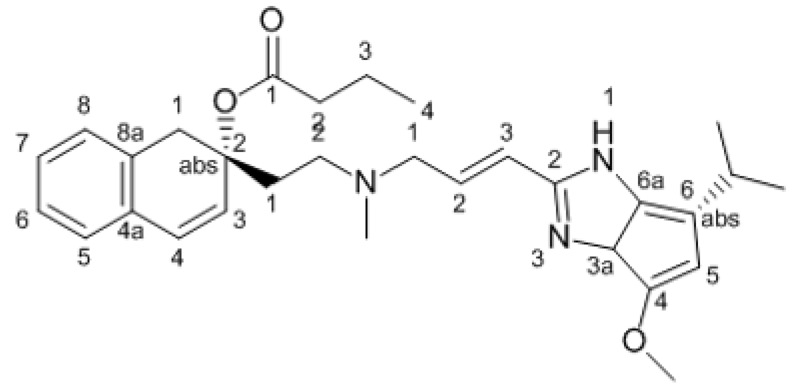

(2R)-2-(2-((E-3-(6-isopropyl-4-methoxy-1,3a-dihydrocyclopental[d]imidazole-2-yl)allyl)(methyl)amino)ethyl)-1,2-dihydronaphthalen-2-yl butyrate

PSA: 63.16

ALogP: 4.4338

Stereo Center Count: 2

Hydrogen Acceptor Count: 6

Hydrogen Donor Count: 1

Composition: C: 73.6% H: 8% N: 8.6% O: 9.8%

Formula Weight: 489.64896

Exact Mass: 489.299142138

Molecular Formula: $C_{30}H_{39}N_3O_3$

Supplementary Figure S9

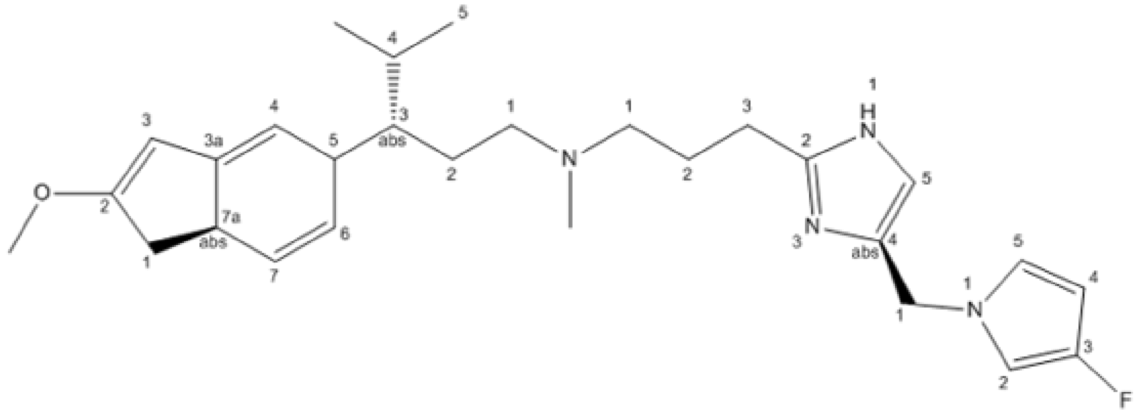

3S)-N-(3-(4-((3-fluoro-1H-pyrrol-1-yl)methyl)-1H-imidazol-2-yl)propyl)-3-((7aR)-2-methoxy-5,7a-dihydro-1H-inden-5-yl)-N,4-dimethylpentan-1-amine

PSA: 46.08

ALogP: 4.85259999999999

Stereo Center Count: 3

Hydrogen Acceptor Count: 3

Hydrogen Donor Count: 1

Composition: C: 72.1% H: 8.4% F: 4.1% N: 12% O: 3.4%

Formula Weight: 466.63386

Exact Mass: 466.310790108

Molecular Formula: $C_{28}H_{39}FN_4O$

Supplementary Figure S10

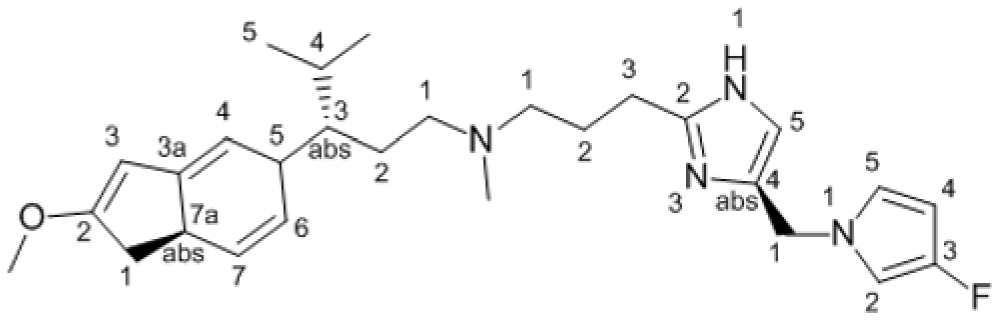

(3S)-N-(3-(4-((3-fluoro-1H-pyrrol-1-yl)methyl)-1H-imidazol-2-yl)propyl)-3-((7aR)-2-methoxy-5,7a-dihydro-1H-inden-5-yl)-N,4-dimethylpentan-1-amine

PSA: 46.08

ALogP: 4.85259999999999

Stereo Center Count: 3

Hydrogen Acceptor Count: 3

Hydrogen Donor Count: 1

Composition: C: 72.1% H: 8.4% F: 4.1% N: 12% O: 3.4%

Formula Weight: 466.63386

Exact Mass: 466.310790108

Molecular Formula: $C_{28}H_{39}FN_4O$

Supplementary Figure S11

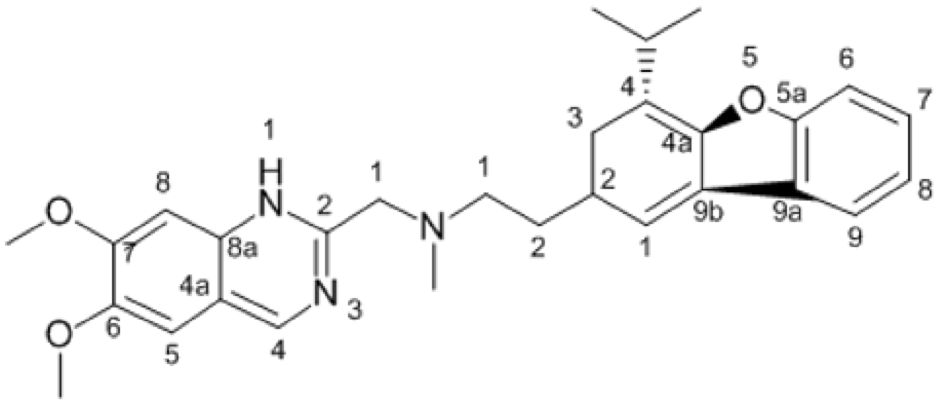

N-((6,7-dimethoxy-1,8a-dihydroquinazolin-2-yl)methyl)-2-(4-isoproyl-2,3-dihydrodibenzo[b,d]furan-2-yl)-N-methylethan-1-amine

PSA: 55.31

ALogP: 3.2038

Stereo Center Count: 2

Hydrogen Acceptor Count: 6

Hydrogen Donor Count: 1

Composition: C: 73.5% H: 7.4% N: 8.9% O: 10.1%

Formula Weight: 473.6065

Exact Mass: 473.26784201

Molecular Formula: $C_{29}H_{35}N_3O_3$

Supplementary Figure S12

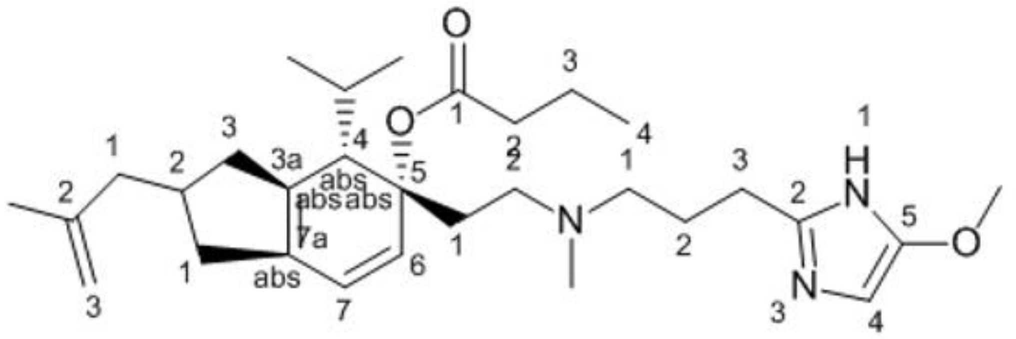

(4S,5R)-4-isopropyl-5-(2-((3-(5-methoxy-1H-imidazol-2-yl)propyl)(methy-amino)ethyl)-2-(2-methylallyl)-2,3,4,5-tetrahydro-1H-iden-5-yl butyrate

PSA: 67.44

ALogP: 5.87169999999999

Stereo Center Count: 3

Hydrogen Acceptor Count: 5

Hydrogen Donor Count: 1

Composition: C: 72.4% H: 9.5% N: 8.4% O: 9.6%

Formula Weight: 497.71248

Exact Mass: 497.361742394

Molecular Formula: $C_{30}H_{47}N_3O_3$

Supplementary Figure S13

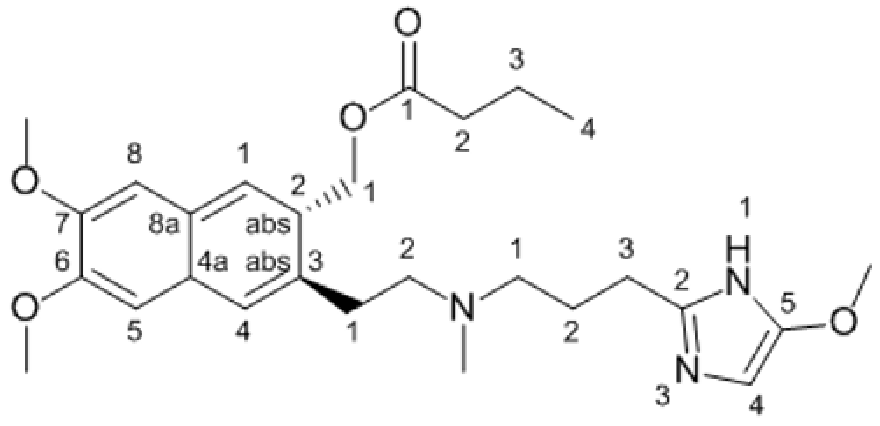

((2S)-6,7-dimethoxy-3-(2-((3-(5-methoxy-1H-imidazol-2-yl)propyl)(methyl)amino)ethyl)-2,4a-dihydronaphthalen-2-yl)methyl butyrate

PSA: 85.91

ALogP: 2.8659

Stereo Center Count: 2

Hydrogen Acceptor Count: 7

Hydrogen Donor Count: 1

Composition: C: 66.8% H: 8.1% N: 8.7% O: 16.5%

Formula Weight: 485.61566

Exact Mass: 485.288971378

Molecular Formula: $C_{27}H_{39}N_3O_5$

Supplementary Figure S14

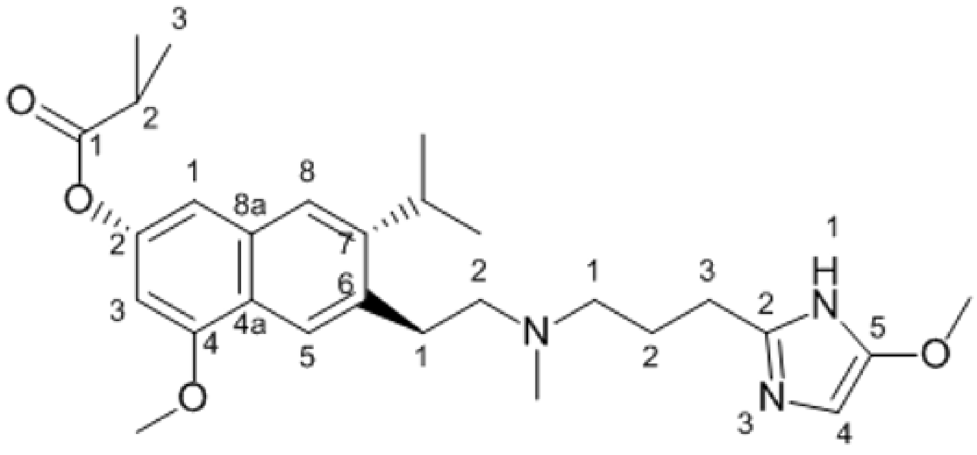

7-isopropyl-4-methoxy-6-(2-((3-(5-methoxy-1H-imidazol-2-yl)propyl)(methyl)amino)ethyl)naphthalen-2-yl isobutyrate

PSA: 76.68

ALogP: 5.5496

Stereo Center Count: 0

Hydrogen Acceptor Count: 6

Hydrogen Donor Count: 1

Composition: C: 69.8% H: 8.2% N: 8.7% O: 13.3%

Formula Weight: 481.62696

Exact Mass: 481.294056758

Molecular Formula: $C_{28}H_{39}N_3O_4$

Supplementary Figure S15

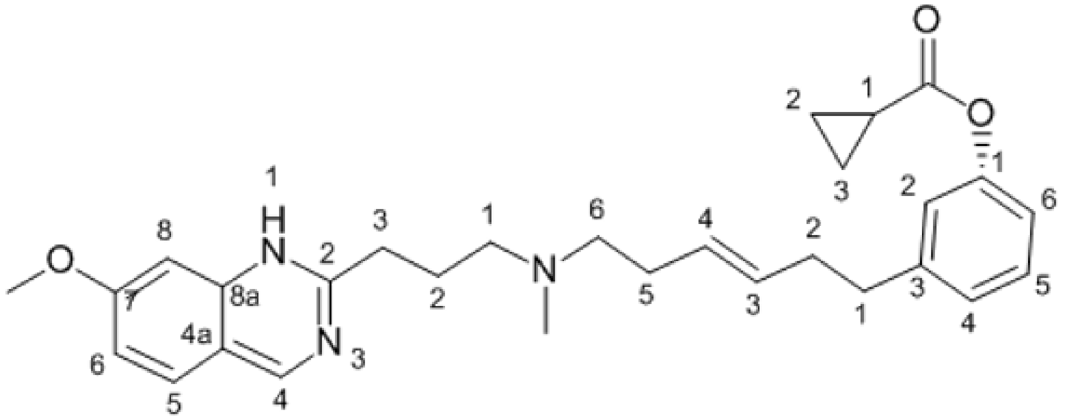

(E)-3-(6-((3-(7-methoxy-1,8a-dihydroquinazolin-2-yl)propyl)(methyl)amino)hex-3-en-1-yl)phenylcyclopropanecarboxylate

PSA: 63.16

ALogP: 4.1399

Stereo Center Count: 1

Hydrogen Acceptor Count: 6

Hydrogen Donor Count: 1

Composition: C: 73.2% H: 7.8% N: 8.8% O: 10.1%

Formula Weight: 475.62238

Exact Mass: 475.283492074

Molecular Formula: $C_{29}H_{37}N_3O_3$

Supplementary Figure S16

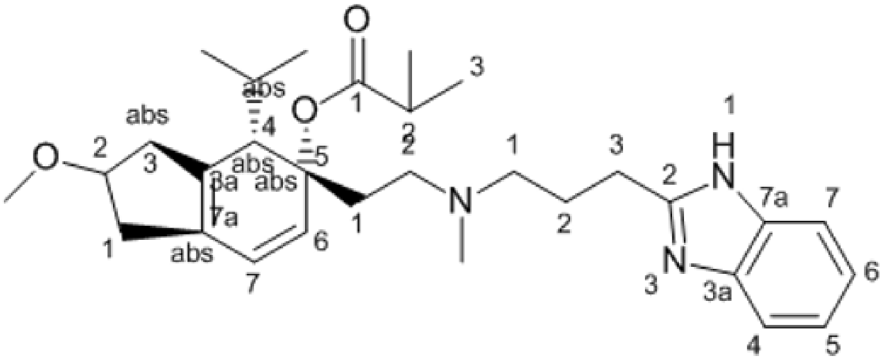

(4S,5R)-5-(2-((3-(1H-benzo[d]imidazole-2-yl)propyl)(methyl)amino)ethyl)-4-isopropyl-2-methoxy-2,3,4,5-tetrahydro-1H-inden-5-yl isobutyrate

PSA: 67.45

ALogP: 5.3499

Stereo Center Count: 3

Hydrogen Acceptor Count: 5

Hydrogen Donor Count: 1

Composition: C: 73% H: 8.8% N: 8.5% O: 9.7%

Formula Weight: 493.68072

Exact Mass: 493.330442266

Molecular Formula: $C_{30}H_{43}N_3O_3$

Supplementary Figure S17

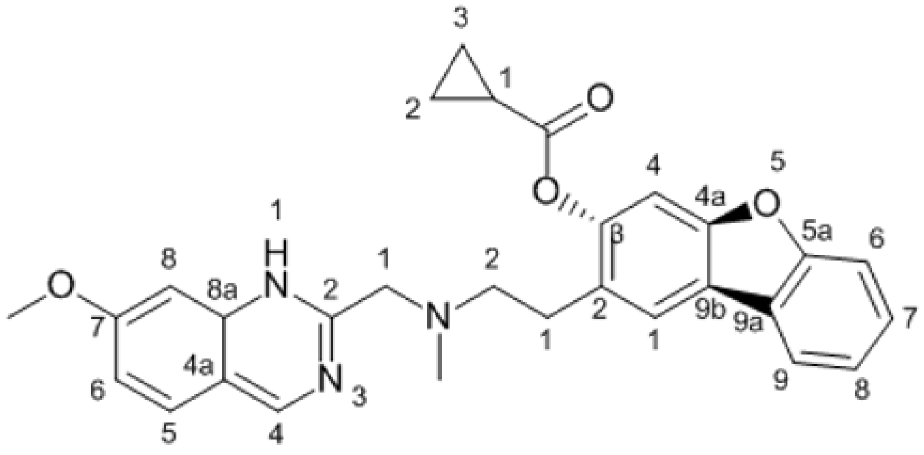

2-(2-(((7-methoxy-1,8a-dihydroquinazolin-2-yl)methyl)(methyl)amino)ethyl)dibenzo[b,d]furan-3-yl cyclopropanecarboxylate

PSA: 76.3

ALogP: 3.6937

Stereo Center Count: 1

Hydrogen Acceptor Count: 6

Hydrogen Donor Count: 1

Composition: C: 72% H: 6% N: 8.7% O: 13.2%

Formula Weight: 483.55826

Exact Mass: 483.215806438

Molecular Formula: $C_{29}H_{29}N_3O_4$

Reference

1. Bezprozvanny, I. and R. Tsien, *Voltage-dependent blockade of diverse types of voltage-gated Ca2+ channels expressed in Xenopus oocytes by the Ca2+ channel antagonist mibefradil (Ro 40-5967).* Molecular pharmacology, 1995. **48**(3): p. 540-549.
2. Huang, L., et al., *NNC 55-0396 [(1S, 2S)-2-(2-(N-[(3-benzimidazol-2-yl) propyl]-N-methylamino) ethyl)-6-fluoro-1, 2, 3, 4-tetrahydro-1-isopropyl-2-naphtyl cyclopropanecarboxylate dihydrochloride]: a new selective inhibitor of T-type calcium channels.* Journal of Pharmacology and Experimental Therapeutics, 2004. **309**(1): p. 193-199.
3. Wu, S., et al., *A mibefradil metabolite is a potent intracellular blocker of L-type Ca2+ currents in pancreatic β-cells.* Journal of Pharmacology and Experimental Therapeutics, 2000. **292**(3): p. 939-943.
4. Heady, T.N., et al., *Molecular pharmacology of T-type Ca2+ channels.* Jpn J Pharmacol, 2001. **85**(4): p. 339-50.
5. Li, M., et al., *Towards selective antagonists of T-type calcium channels: design, characterization and potential applications of NNC 55-0396.* Cardiovascular drug reviews, 2005. **23**(2): p. 173-196.
6. Singh, A., et al., *The transient receptor potential channel antagonist SKF96365 is a potent blocker of low-voltage-activated T-type calcium channels.* British journal of pharmacology, 2010. **160**(6): p. 1464-1475.
7. Chen, Y., W.D. Parker, and K. Wang, *The role of T-type calcium channel genes in absence seizures.* Front Neurol, 2014. **5**: p. 45.
